# 

**Published:** 1871-09

**Authors:** 


### Table of Contents.

ORIGINAL COMMUNICATIONS.

A Case of Typhoid Fever, with Thoughts and Reflections on the Leading Features of the Pathology and Treatment of Low Continued Fevers. By Ch. Raushenberg, M.D., Atlanta, Georgia.. 303

Resume of Recent Discoveries and Improvements in Medicine. By Wm. Abram Love, M. D., Atlanta, Georgia.................. 313

Atlanta Academy of Medicine. Reported by J. T. Johnson, M.D. 320

Atlanta Academy of Medicine. Reported by Edwin P. Ingraham, M.D.......................................... 330

Indian Spring—Location, Properties and Medicinal Virtues of the Water. By J. T. Banks, M. D.............................. 333

Case of Cerebro-Spinal Meningitis Successfully Treated. By Wm. O'Daniel, M.D., Twiggs County, Georgia.................... 336

Strangulated Hernia—Removal of Three and a Half Ounces of Omentum. By W. F. Westmoreland, M D., Professor of Surgery in Atlanta Medical College............................ 338

SELECTED ARTICLES.

Report on Surgery for the Semi-Annual Session of the Æsculapian Society of the Wabash Valley, May 25, 1871. By Leon J. Willien, M.D., Effingham, Illinois............................ 340

Ice in the Rectum in Retention of Urine........................ 350

EDITORIAL AND MISCELLANEOUS.

Editorial Correspondence.................................... 351

Our Journal.................................................. 355

Progress..................................................... 356

Atlanta Academy of Medicine.................................. 357

New York Correspondence...................................... 357

The Late Dr. D. C. O'Keefe.................................... 361

